# Study of Forming Performance and Characterization of DLP 3D Printed Parts

**DOI:** 10.3390/ma16103847

**Published:** 2023-05-19

**Authors:** Ting Jiang, Bo Yan, Minzheng Jiang, Buguang Xu, Sheng Gao, Yi Xu, Yueqiang Yu, Tingang Ma, Tao Qin

**Affiliations:** 1 College of Mechanical Science and Engineering, Northeast Petroleum University, Daqing 163318, China; jiangting1112@163.com (T.J.); 18710605661@163.com (B.Y.); yuyaoqiang.1228@163.com (Y.Y.); mtinga0@163.com (T.M.); qsg9772@163.com (T.Q.); 2 Key Laboratory of Petroleum Mechanical Engineering of Heilongjiang Province, Daqing 163318, China; 3 Ningbo Runyes Medical Instrument Co., Ltd., Ningbo 315300, China; xu202147@163.com (B.X.); xuyi__2021@163.com (Y.X.); 4 Research and Development Center of 3D Printing Material and Technology, Northeast Forestry University, Harbin 150040, China

**Keywords:** 3D printing, DLP technology, layer thickness, mechanical property, characterization

## Abstract

In order to explore the effect of printing parameter configurations on the forming performance of Digital Light Processing (DLP) 3D printed samples, printing experiments were carried out on the enhanced adhesion and efficient demolding of DLP 3D printing devices. The molding accuracy and mechanical properties of the printed samples with different thickness configurations were tested. The test results show that when the layer thickness increases from 0.02 mm to 0.22 mm, the dimensional accuracy in the X and Y directions increases first and then decreases, while the dimensional accuracy in the Z direction decreases, and the dimensional accuracy is the highest when the layer thickness is 0.1 mm. The mechanical properties of the samples decline with an increasing layer thickness of the samples. The mechanical properties of the 0.08 mm layer thickness are the best, and the tensile, bending, and impact properties are 22.86 Mpa, 48.4 Mpa, and 35.467 KJ/m^2^, respectively. Under the condition of ensuring molding accuracy, the optimal layer thickness of the printing device is determined to be 0.1 mm. The analysis of the section morphology of samples with different thicknesses illustrates that the fracture of the sample is a river-like brittle fracture, and there are no defects such as pores in the section of samples.

## 1. Introduction

Additive manufacturing is a kind of manufacturing method that stacks raw materials in layers [1]. It can manufacture complex parts quickly and accurately to achieve “free manufacturing”, greatly reduce the processing procedures, and shorten the processing cycle [2]. Three-dimensional printing technology mainly includes Fused Deposition Modeling (FDM) [3], Selective Laser Sintering (SLS) [4], Laminated Object Manufacturing (LOM) [5], and Digital Light Processing (DLP) [6]. It is used to rapidly manufacture parts directly through three-dimensional data information and is applied in many engineering fields [7]. Stereo Lithography Apparatus (SLA) was proposed by Dr. Charles W. Hull [8] in a patent in 1984. The material is a liquid photosensitive resin, which undergoes a polymerization reaction and solidifies when irradiated with UV light of a specific wavelength [9]. With the development of photocuring technology, it can be divided into three categories at present: Stereo Lithography Apparatus (SLA), Digital Light Processing (DLP), Liquid Crystal Display (LCD), etc. Their difference lies in the use of different light sources [10]. Compared with Stereo Lithography Apparatus (SLA) and Liquid Crystal Display (LCD), the principle of DLP 3D printing technology is the processing method of photosensitive resin and stacking layer by layer through the way of surface forming [11]. It can print and solidify a single layer of liquid resin in just a few seconds, thus printing sample models faster with a higher processing accuracy [12]. Therefore, it is often applied in the fields of dentistry [13] and jewelry [14].

Before the printing task, 3D printing technology needs to set the layer thickness of the model on the slicing software. With the other parameters unchanged, the layer thickness determines the overall printing time of the model. Many factors affect the performance of printing models, such as exposure time, printing direction, power density of the light source, etc., but the thickness is probably the most important factor in a given printed condition [15]. The layer’s thickness constitutes the lamination direction of 3D printing, which is the weakest construction direction in the printing process and also the focus of researchers. Ouassil et al. [16] studied the influence of the Z-direction porosity on the tensile properties of polyether imide (PEI) and determined the optimal parameters. Favero et al. [17] believed that the accuracy of the printed model declined as the monolayer thickness was decreased by SLA, which is also applicable to DLP technology. They found that when the layer thickness changed from 0.1 mm to 0.25 mm, the mean overall deviation decreased by 22.9%. According to the conclusion, the single-layer thickness should be set to be larger in order to reduce the overall printing time. Zhang et al. [18] found that as the layer thickness decreased from 0.10 mm to 0.05 mm, the accuracy of the DLP 3D printing model increased. The average absolute deviation fell by 59%. Kazemi and Rahimi et al. [19] found that, compared with other parameters, the layer thickness has a greater impact on tensile strength, and the optimum layer thickness was determined to be 0.15 mm. Wang et al. [20] studied the surface accuracy and dimensional accuracy of samples under different layer thicknesses. It was found that when the layer thickness was too small, the ‘Z’ axis appeared surplus phenomenon, and the layer thickness had little effect on the horizontal dimensional accuracy. When the layer thickness increases from 0.08 mm to 0.1 mm, the tensile strength decreases from 49.98 MPa to 46.62 MPa. Saini et al. [21] used SLA technology to print samples with different printing angles, studied their mechanical properties, and obtained the best mechanical properties of the samples with different printing angles. It can be seen that the setting of the layer thickness is still controversial, and the success of the DLP 3D printing process largely depends on the appropriate parameters and the different parameter settings of different machines. We need to explore the appropriate layer thickness parameter settings in the 3D printing process to achieve the best performance of the printing device.

Based on the problems of weak model adhesion and low demolding efficiency in the process of DLP 3D printing, Jiang et al. [22] designed a DLP 3D printing device that integrates efficient demolding and reinforced adhesion. However, the influence of the change in the “layer thickness” parameter on manufactured samples by this device needs to be studied in depth. Therefore, this work measures and plots the dimensional accuracy and mechanical property curves between samples with different thicknesses. By analyzing the change mechanism of the micromorphology of samples with different layer thicknesses, the variation rule of the molding properties of samples with different thicknesses and the appropriate layer thickness parameter setting of the printer were obtained to provide a reference for the layer thickness setting of the stereolithography 3D printing equipment.

## 2. Printing Experiment

### 2.1. Printing Principle

**Figure 1:**
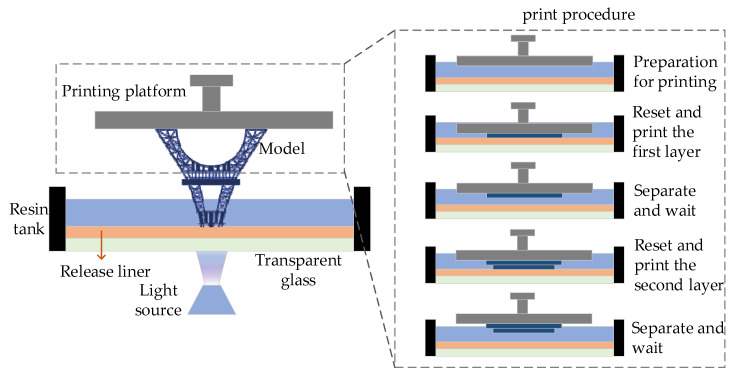
Constrained liquid surface formation schematic diagram.

According to the position of the projection device, the DLP 3D printing device included a constrained liquid-level 3D printing device [23] as well as a free liquid-level 3D printing device [24]. Compared with the free liquid-level forming method, the constrained liquid-level forming method does not require a scraping device, so the printing speed is faster. Its printing height of the model is not affected by the height of the resin tank, more resin material is saved [25], and the solidified part of the constrained liquid-level forming method is limited to the part between the formed part and the resin tank, which is not easy to deform, warp, or cause any other problems. As shown in Figure 1, the principle of DLP is the process of printing the solid model layer by layer using the polymerization and curing principles of photosensitive resin by way of surface projection of layered images of the model. First, the 3D model is sliced in the slicing software, imported into the device, and the resin is poured into the resin tank to start printing. The platform moves down to the resin tank, and the DLP projection device projects the sliced images onto the bottom of the resin tank to solidify the formed layer. Secondly, the forming layer attaches to the upward printing platform. Then, the printing platform prints down the second layer, and so on, layer by layer. Finally, the formed model can be obtained [26].

### 2.2. Experimental Equipment

**Figure 2:**
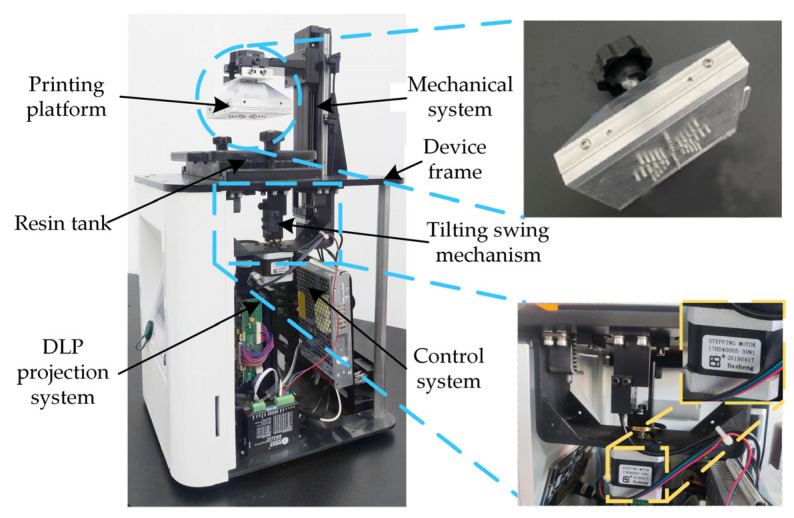
DLP 3D printer.

In this experiment, the DLP 3D printer (Figure 2) designed by us includes a mechanical motion system, a DLP projection system, a hardware, and software control system, etc. Unlike the traditional DLP 3D printing device, its printing platform and tilting swing mechanism can not only effectively solve the problem of model shedding caused by excessive separation force in the printing process, but they can also solve the problem of model stripping difficulties after printing [22]. Table 1 shows the parameters of the DLP 3D printer. After testing, the printer can realize the basic printing function, and the overall performance of the printer is stable. The forming accuracy test and performance experiments were designed to test the effect of a single-layer thickness on the forming performance.

### 2.3. Test and Characterization

The experiment was carried out using the ordinary rigid resin produced by Shenzhen Yidimu Intelligent Technology Co., Ltd., Shenzhen, China. The main components of the resin material include polyurethane acrylate (PUA), trimethylol propane triacrylate (TMPTA), modified acrylate, an accessory ingredient, and a photoinitiator, etc.

**Figure 3:**
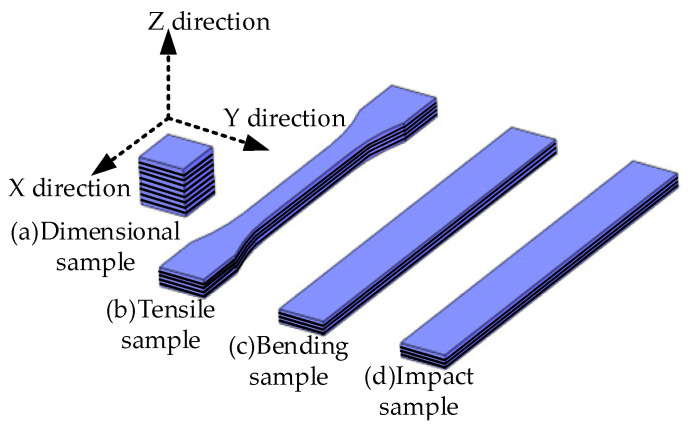
Schematic diagram of printing direction.

The forming accuracy analysis: The 10 mm × 10 mm × 10 mm cube model was set to 0.02 mm, 0.04 mm, 0.06 mm, etc., for printing until the printing was unqualified. Figure 3a shows the X, Y, and Z directions of the sample. When the printing work was completed, the sample was removed from the printing platform, soaked in anhydrous ethanol, cleaned to remove any residual resin, and then dried. The vernier caliper was used to measure the actual size of the sample in three directions, and the average value of each direction was measured three times at different positions. Then, the actual size and dimensional accuracy change curves are drawn. Formula (1) for calculating the dimensional accuracy is as follows:

(1) $$\delta =1-\frac{\left|L_{0}-\right.\left.L\right|}{L_{0}}\times 100\%$$

where *δ* is the dimensional accuracy; *L*_0_ is the standard size (mm); and *L* is the measured size (mm).

The tensile strength test: Figure 3b shows the tensile sample model. The tensile sample models with different thicknesses were sliced and printed. The tensile test was performed on the universal tensile tester of model CMT5105, and the tensile test was performed according to the ISO 527-2:1993 standard at a tensile speed of 1 mm/min. There are five tensile samples, which were measured to obtain the average value.

The bending property test: Figure 3c shows the bending sample model. The bending sample models with different layer thicknesses were sliced and printed, and the experiment was carried out on the universal mechanical testing machine of INSTRON 3343. The bending strength was tested according to the ISO 178:2001 standard. The size of the bending sample was 80 mm × 10 mm × 4 mm, and the crosshead speed was 2 mm/min. There were five bending samples, which were measured to obtain the average value.

The impact performance test: Figure 3d shows the bending sample model. Impact sample models with different thicknesses were sliced and printed, and experiments were carried out on the INSTRON 9250HV pendulum impact machine. The impact strength was tested according to the ISO 179-2:1997 standard. The impact sample size was 75 mm × 10 mm × 3 mm; the sample type was not notched; the crosshead speed was 2 mm/min; and the pendulum impact energy was 4 J. There were five impact samples, which were measured to obtain the average value.

Scanning electron microscope: Samples with different thickness configurations were soaked in anhydrous ethanol and dried. The tensile section of the sample was scanned by the TESCAN MIRA LMS scanning electron microscope. Since the sample material was a photosensitive resin curing part with poor conductivity, a sputtering coater instrument, Quorum SC7620, was used to spray gold on its section, and then the sample section was scanned to obtain the microscopic morphology SEM diagram of the sample section.

## 3. Results and Discussion

### 3.1. Forming Accuracy

As shown in Table 2, the room temperature was set to 20 °C, the single-layer exposure time of the device was 3.5 s, the bottom quantity was one layer, the power density was 50 mW/cm^2^, and the bottom exposure time was 28 s. On the premise of keeping the other parameters unchanged, the parameter configuration of the single-layer thickness was changed to explore the variation rule of the single-layer thickness on the size and dimensional accuracy of samples in the X, Y, and Z directions.

**Figure 4:**
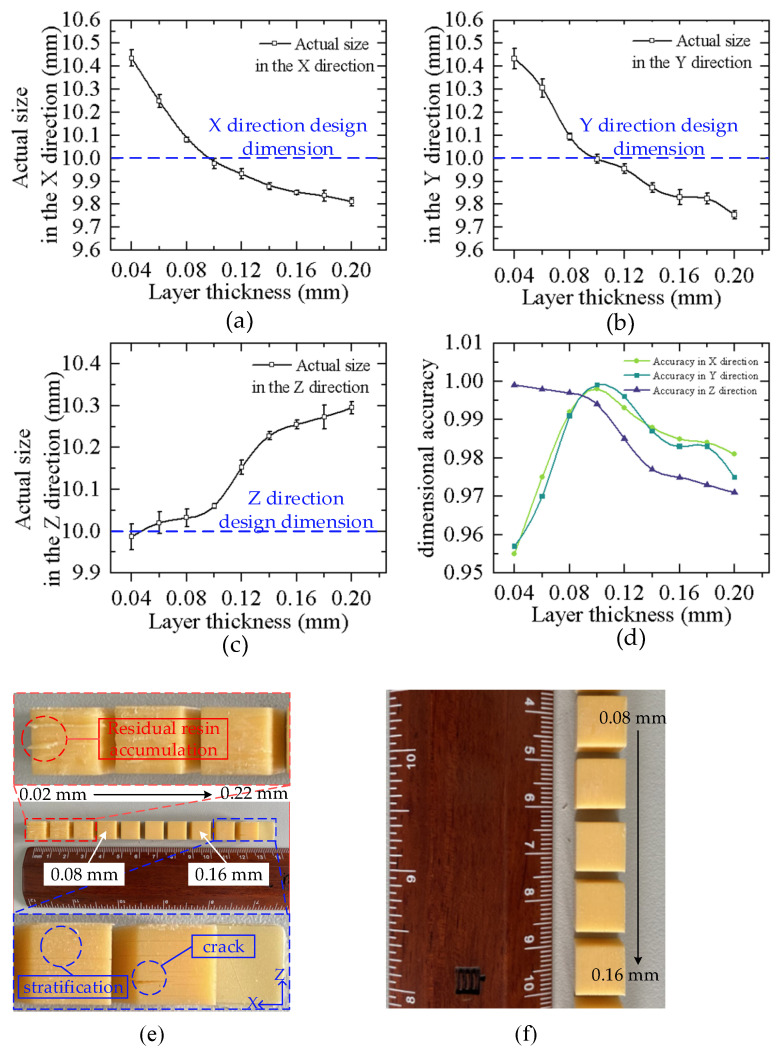
Curve of actual size and dimensional accuracy. (**a**) The actual size and deviation curve in X direction. (**b**) The actual size and deviation curve in Y direction. (**c**) The actual size and deviation curve in Z direction; (**d**) The variation curve of the dimensional accuracy in three directions. (**e**) The printing diagram of the flaw sample. (**f**) The printing samples.

Figure 4 depicts the actual size and dimensional accuracy curves of different thickness models in three directions. Figure 4a–c illustrates that an increase in thickness of a single layer causes the actual dimensions in the X and Y directions of the samples to decrease and those in the Z directions to increase. Figure 4d shows that the dimensional accuracy of the samples in the X and Y directions climbs up and then declines with an increasing layer thickness, while the dimensional accuracy in the Z direction decreases with an increasing layer thickness. With a layer thickness of 0.1 mm, the actual size in the X and Y directions is closest to the design size, and the overall dimensional accuracy is the highest. Figure 4e demonstrates the printing results of the samples. The samples are placed in the Z-axis stacking direction, with the layer thickness successively ranging from 0.02 mm to 0.22 mm. The residual resin accumulation occurs at the edge of the sample when the layer thickness is less than 0.08 mm, and cracks appear in the direction of the Z-axis when the layer thickness is more than 0.16 mm. In Figure 4f, the sample with a layer thickness of 0.08–0.16 mm has a smooth surface and good molding quality. This is because the curing process of photosensitive resins follows the Beer–Lambert law, as shown in Formula (2):

(2) $$C_{d}=D_{p}\ln\frac{E}{E_{c}}$$

where *C_d_* is curing depth; *D_p_* is the propagation depth of UV light; *E* is the exposure energy; and *E_c_* is the critical exposure.

The curing process of photosensitive resin can be divided into two stages, namely the pre-curing stage and the full curing stage. In the pre-curing stage, the photosensitive resin liquid absorbs UV energy continuously so that it reaches a critical state and the liquid resin begins to cure. In the full curing stage, the liquid phase of the photosensitive resin is completely transformed into a solid. When the energy absorbed by the photosensitive resin reaches the pre-curing stage but not the full curing stage, it belongs to the under-curing state. When the energy absorbed by the photosensitive resin reaches the curing stage and continues to absorb energy, it will enter the over-curing state. By increasing the thickness of the layer, the energy absorbed by the full curing of the single-layer resin increases. When the layer thickness is small, the curing degree of the same exposure time is higher than that of the larger layer thickness. The sample has reached the stage of full curing and entered the state of over-curing. Excessive exposure to energy spillover will make the actual size in the X and Y directions larger than the designed size, and residual accumulation phenomenon of resin curing at the edge of the sample. Excessive exposure to energy leads to over-curing of the sample in the molding process, which makes the actual size of the X and Y directions larger than the designed size. Excessive exposure to energy spillover will cause residual accumulation of resin curing at the edge of the sample. When the layer thickness is greater than 0.16 mm, the step pattern in the stacking direction of the sample is obviously visible, and the accuracy of the model decreases. When the layer thickness is 0.20 mm, cracks appear in the stacking direction, because the thickness of the single layer is too large and the exposure energy is insufficient, so the sample does not reach the full curing stage and stays in the under-curing state. Too little energy exposure makes the actual size in the X and Y directions smaller than the designed size, and weakens the binding force of the sample stacking interface, resulting in cracks, and the dimensional accuracy, shape accuracy, and surface roughness cannot be guaranteed. Reductions in the layer thickness cause an increase in the number of slicing layers, so the dimensional accuracy in the Z direction becomes higher with decreases in the layer thickness. According to the printed results, the mechanical properties of the samples with thicknesses from 0.08 mm to 0.16 mm were tested.

### 3.2. Microstructure

**Figure 5:**
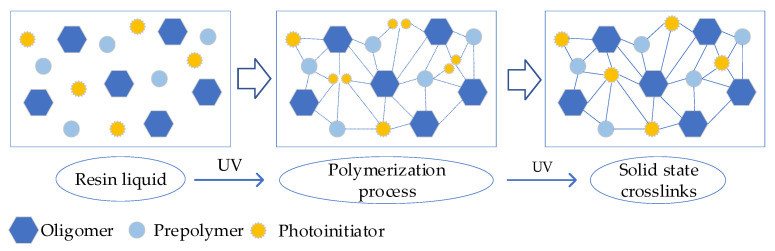
Curing mechanism of photopolymer.

Figure 5 shows the curing mechanism of the photopolymer. The reaction of the liquid resin is mainly composed of oligomer molecules under the action of van der Waals force. While in the process of photocuring, covalent bonds are generated through the C=C reaction, which shortens the distance between atoms and rearranges them to form a tighter 3D network structure, thus causing curing and volume shrinkage. The photocuring material system includes a free radical polymerization system and a cationic polymerization system due to the different principles of the photoinitiator. The reactions involved in this experiment belong to a free radical polymerization system that mainly consists of chain initiation, chain growth, chain termination, and chain transfer processes. Formulas (3)–(8) include the typical reactions in a photopolymerization system [27], wherein the typical reactants include initiator In, free radical R* produced by initiator, monomer *M*, etc. In the first step, the initiator molecule generates two free radicals under ultraviolet irradiation, and the reaction rate is represented by *k_d_*:

(3) $$In\overset{K_{d}}{\to }2R^{*}$$

The free radical then reacts freely with the polymer monomer *M*, thereby triggering a polymer chain to form a polymer chain. The reaction rate is expressed by *k_p_*:

(4) $$R^{*}+M\overset{k_{p}}{\to }P^{*}$$

Polymer chains propagate by reacting with a double bond of C=C on a monomer *M* or another polymer, and the polymer becomes larger when the polymer chains react with each other:

(5) $$P^{*}+M\overset{k_{p}}{\to }P^{*}$$

The reactive free radical and free radical on the polymer molecule react with the free radical produced by the initiator or the free radical on the chain to form the terminal polymer chains *P_dead_* and *R_dead_*, and the polymerization reaction ends. The reaction rate is given by *k_t_*.

(6) $$R^{*}+R^{*}\overset{k_{t}}{\to }2R_{dead}$$

(7) $$P^{*}+P^{*}\overset{k_{t}}{\to }P_{dead}$$

(8) $$P^{*}+R^{*}\overset{k_{t}}{\to }P_{dead}$$

**Figure 6:**
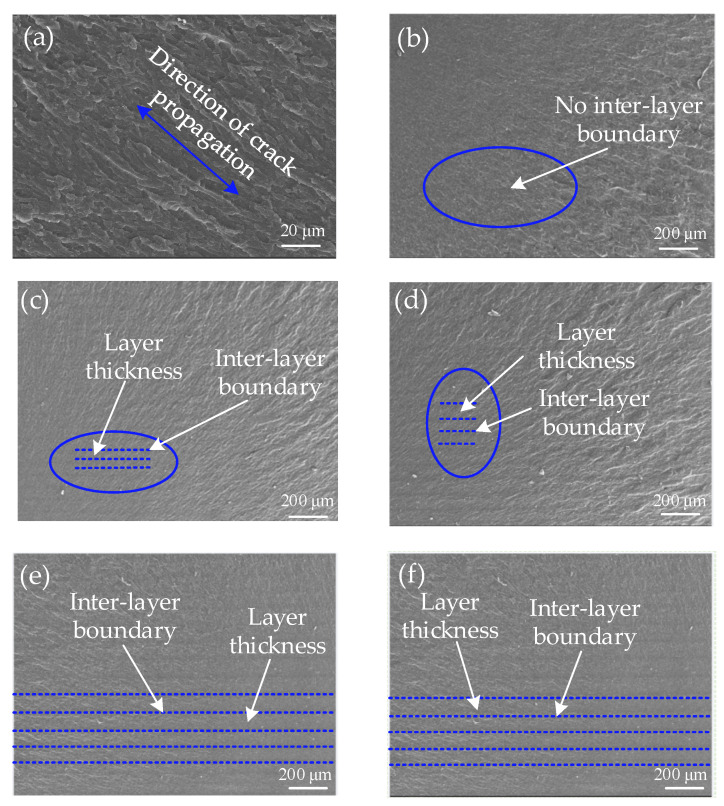
SEM image of tensile fracture of samples. (**a**) High magnification microstructure of tensile fracture of samples. (**b**–**f**) Low magnification microstructure of different printing layer thickness samples.

**Figure 7:**
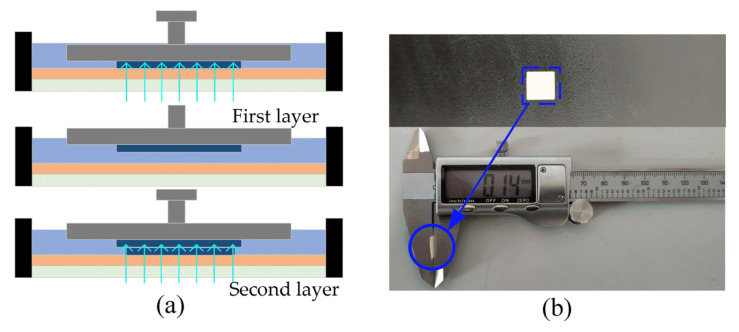
Verification diagram of secondary curing experiment. (**a**) Schematic diagram of secondary curing. (**b**) Schematic diagram of secondary curing experiment.

Figure 6 depicts the SEM image of the tensile fracture of the sample. The tensile fracture mode of all the resin-cured samples is a brittle rupture; the microscopic morphology of the fracture surface is a river-like pattern; and the river-like flow direction is the crack propagation direction of the resin-cured samples. From Figure 6c–f, it can be seen that the stratification of the fracture is in line with the DLP 3D printing lamination principle. The appearance of stratification causes the Z-direction surface of the sample to not be smooth. With the layer thickness of 0.08 mm, the stratification of the resin-cured sample is not observed, but with the increasing layer thickness, the stratification of the resin-cured sample is more and more obvious, and it is most clear when the layer thickness is 0.16 mm. This is because the smaller the layer thickness is, the more UV energy is absorbed by the resin monolayer curing thin layer, the higher the percent conversion of the C=C double bond, the more free radicals exist in the chain growth, and the more thorough the polymerization cross-linking reaction, so the bond between the layers is tighter, and the stratification phenomenon is not obvious. However, when the thickness of the printing layer is larger, the single-layer thin layer needs to absorb more energy, the percent conversion of the C=C double bond is low, the chain growth of free radicals is less, and the polymerization cross-linking reaction is not complete, so the stratification phenomenon between the layers is more obvious. Another reason for this phenomenon may be the secondary curing of the resin sample. In Figure 7a, with a smaller layer thickness, the UV light will penetrate the current curing layer and project to the upper curing thin layer. The C=C double bond will continue to transform, the free radical will continue to grow in chains, the layers will be integrated, and the stratification phenomenon will disappear. On the contrary, the stratification will become more obvious. To investigate the existence of this phenomenon, the actual curing depth of a 10 mm × 10 mm square was tested at room temperature at 20 °C, single-layer exposure time of 3.5 s and a power density of 50 mW/cm^2^. As shown in Figure 7b, the test shows that the curing thickness of the square is 0.14 mm and that of the single layer is thicker than 0.1 mm, indicating that when the thickness of the single layer is small, UV light can be projected through the current thin layer and onto the previous thin layer, resulting in secondary curing of the previous thin layer.

## 4. Mechanical Strength

**Figure 8:**
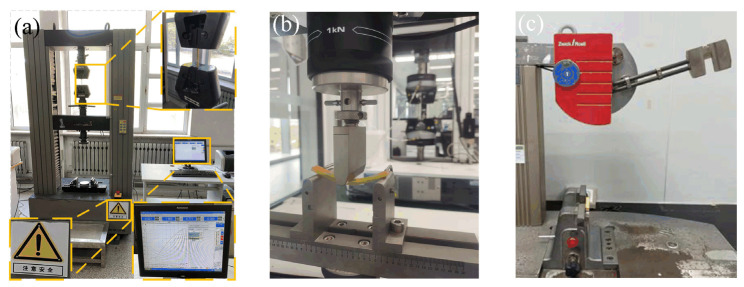
Experimental state diagram of sample (**a**) tensile test, (**b**) bending test, and (**c**) impact test.

**Figure 9:**
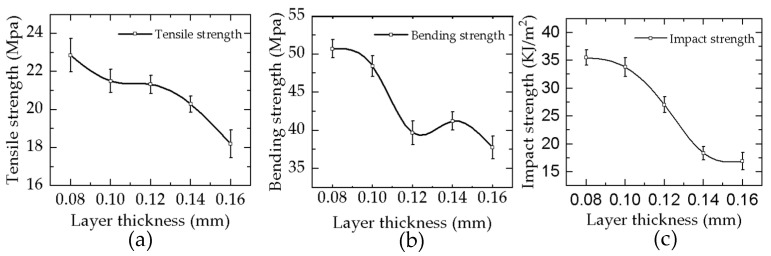
Mechanical properties and deviation curves of samples under different thickness. (**a**) Tensile strength; (**b**) bending strength; (**c**) impact strength.

The layer thickness is a key factor that influences the mechanical properties of the DLP 3D printing samples. Therefore, the mechanical properties of samples with different layers are tested to explore the variation rules of the mechanical properties of samples with different layers. Figure 8a–c depicts the experimental state diagram of the tensile, bending, and impact of the sample. Figure 9a–c depicts the curves of the tensile, bending, and impact strengths of photocuring 3D printing samples configured with different thickness layers. Figure 9 illustrates that the variation trends of the tensile, bending, and impact strengths of tensile samples are roughly the same. These strengths of the samples all decline with an increasing monolayer thickness and reach the best results when the thickness of the sample is 0.08 mm, and their values are 22.86 Mpa, 48.4 Mpa, and 35.467 KJ/m^2^, respectively. The cross-section microstructure of the sample shows that the stratification phenomenon of the sample becomes more obvious when the layer thickness increases. The stratification phenomenon leads to a smaller bonding force between the layers, so its mechanical properties become worse. According to the Beer–Lambert law and the reaction mechanism of the photopolymer, as the single-layer resin liquid continues to absorb UV energy, it will gradually reach the full curing stage. When the layer thickness is 0.08 mm, its curing degree is lower than that of the sample with a layer thickness of 0.16 mm. When the layer thickness is small, the single-layer liquid resin obtains more exposure energy, the conversion rate of the C=C double bond is higher, the chain growth of the free radicals is greater, the reaction of the liquid resin is more thorough, and its reaction state is closer to the full curing stage. Therefore, each layer of the polymer molecules is more closely bound to each other, so their mechanical properties are better. Moreover, more energy will make the resin layer more dense. Under the action of secondary curing, the resin layer will bond more closely, the inter-layer bonding force is larger, the stratification phenomenon gradually disappears, and more energy is needed to destroy the bond between them, so the tensile, bending, and impact strengths are greater. In Figure 9a, when the layer thickness is more than 0.12 mm, the tensile property decreases significantly; when the layer thickness is 0.16 mm, the tensile strength of the sample decreases by 14.6% compared with that of the 0.12 mm samples. In Figure 9b, when the layer thickness is greater than 0.10 mm, the bending strength declines rapidly; when the layer thickness is 0.16 mm, the bending strength decreases by 17.0% relative to the layer thickness of 0.10 mm. In Figure 9c, when the layer thickness is greater than 0.12 mm, the impact strength decreases rapidly; when the layer thickness is 0.16 mm, the impact strength of the sample with a 0.12 mm layer thickness decreases by 37.4%. In summary, the mechanical properties of samples with thicknesses of 0.08 mm, 0.10 mm, and 0.12 mm are the best, and their mechanical properties decline in turn.

## 5. Conclusions

The effect of different thickness layers on the forming properties of photocuring DLP 3D printing samples was explored. The experimental samples were manufactured using an efficient demolding and enhanced adhesion photocuring DLP 3D printer. The dimensional accuracy and mechanical properties of the samples with different thickness layers were experimentally studied, and the following specific conclusions were drawn:

(1) The actual size and accuracy of the X, Y, and Z directions of the samples with different thicknesses were measured, and it was found that the layer thickness had a great influence on the size of the X, Y, and Z directions of the samples. The larger the layer’s thickness, the smaller the actual size of the X and Y directions, and the larger the actual size of the Z direction. When the layer thickness is small, the sample will exhibit an over-curing phenomenon, which makes the actual size of the samples larger than the design size, and there will be residual resin accumulation. When the thickness of the sample is small, the under-curing phenomenon occurs, which makes the actual size smaller than the design size, and cracks appear on the sample surface. The dimensional accuracy of the samples in the X and Y directions increases first and then decreases, but decreases in the Z direction. When the layer thickness is 0.1 mm, the overall dimensional accuracy of the samples is the best.
(2) Through the analysis of the section morphology of samples with different thicknesses, it was found that the fracture of the sample was a river-like brittle fracture, and there were no defects such as pores in the sections of the samples with different thicknesses. When the layer thickness is larger, the microstratification is more obvious. When the layer thickness is 0.16 mm, the microstratification is the most obvious. The reason for this phenomenon follows the Beer–Lambert law and may be caused by the secondary curing of the sample layer.
(3) Through the analysis of the mechanical properties of the samples with different thicknesses, the tensile strength, bending strength, and impact strength of the printing samples all declined as the layer thickness of the printed samples increased, and the best result was achieved with a layer thickness of 0.08 mm. When the layer thickness was greater than 0.12 mm, the mechanical properties of the samples declined. The dimensional accuracy of the samples with a thickness of 0.1 mm was the highest, while the mechanical properties of the samples with a thickness of 0.08 mm were not much different from those of 0.1 mm. On the premise of ensuring the high dimensional accuracy of the DLP 3D printing samples, based on the analysis of the micromorphology and mechanical properties of the samples, it can be seen that the optimal layer thickness parameter is 0.1 mm, as this causes the highest overall dimensional accuracy of the samples and its mechanical properties are better.

## Figures and Tables

**Table 1:** Device parameters.

Device Parameters	Value
Printer size	320 mm × 300 mm × 585 mm
Input parameter	220 V AC, 50 HZ
Maximum print size	89.6 mm × 56 mm × 95 mm
Power	65 W
Printing speed	40 mm/h

**Table 2:** Print parameter setting.

Parameters	Value
Indoor temperature	20 °C
Single-layer exposure time	3.5 s
Bottom quantity	1
Power density	50 mW/cm^2^
Bottom exposure time	28 s

## Data Availability

Not applicable.
